# Supplementary material for: Sleep duration irregularity and risk for incident cardiovascular disease in the UK Biobank
Source: medRxiv. 2024 Jul 27:2024.07.26.24311090. Preprint. [Version 1] doi: 10.1101/2024.07.26.24311090 (PMC11302714; doi:10.1101/2024.07.26.24311090)
Supplement: Supplement 1 [file NIHPP2024.07.26.24311090v1-supplement-1.pdf]

Supplemental Table 1. Associations of accelerometer-measured sleep duration irregularity with incident cardiovascular events in the UK Biobank, censored at the start of the COVID-19 pandemic lockdown (March 23, 2020)

	7-day sleep duration standard deviation					Per 1 hour	P-trend
	≤30 min	31-45 min	46-60 min	60-90 min	>90 min		
Major cardiovascular events							
Cases	205	449	393	304	173	1524	
Person-years	69140	140444	119186	89709	50157	468635	
Model 1	Ref	1.15 (0.98, 1.36)	1.28 (1.08, 1.51)	1.42 (1.19, 1.69)	1.51 (1.23, 1.85)	1.24 (1.13, 1.35)	<.0001
Model 2	Ref	1.13 (0.96, 1.34)	1.23 (1.04, 1.46)	1.32 (1.11, 1.58)	1.41 (1.15, 1.72)	1.20 (1.10, 1.31)	<.0001
Model 3	Ref	1.13 (0.96, 1.33)	1.22 (1.03, 1.45)	1.31 (1.09, 1.57)	1.35 (1.10, 1.67)	1.17 (1.07, 1.28)	0.0008
Myocardial infarction							
Cases	109	218	213	160	97	797	
Person-years	69323	140940	119576	90017	50329	470185	
Model 1	Ref	1.05 (0.83, 1.32)	1.29 (1.03, 1.63)	1.38 (1.08, 1.76)	1.54 (1.17, 2.03)	1.30 (1.15, 1.46)	<.0001
Model 2	Ref	1.03 (0.82, 1.30)	1.23 (0.98, 1.55)	1.26 (0.99, 1.62)	1.41 (1.07, 1.86)	1.25 (1.11, 1.41)	0.0003
Model 3	Ref	1.02 (0.81, 1.29)	1.21 (0.96, 1.52)	1.23 (0.96, 1.57)	1.30 (0.98, 1.73)	1.20 (1.06, 1.35)	0.004
Stroke							
Cases	101	235	187	152	81	756	
Person-years	69353	140907	119671	90035	50394	470359	
Model 1	Ref	1.22 (0.97, 1.55)	1.24 (0.98, 1.58)	1.46 (1.14, 1.88)	1.47 (1.10, 1.98)	1.19 (1.04, 1.35)	0.009
Model 2	Ref	1.21 (0.96, 1.52)	1.21 (0.95, 1.54)	1.39 (1.08, 1.79)	1.40 (1.05, 1.89)	1.16 (1.02, 1.33)	0.02
Model 3	Ref	1.21 (0.96, 1.53)	1.21 (0.95, 1.55)	1.40 (1.09, 1.81)	1.41 (1.04, 1.90)	1.16 (1.01, 1.33)	0.03

Model 1: adjusted for age, sex, race, Townsend deprivation index, work schedules, and family history of CVD
Model 2: Model 1 + adjusted for BMI, smoking status, alcohol consumption, diet quality, physical activity, and history of hypertension, dyslipidemia, diabetes and depression
Model 3: Model 2 + adjusted for accelerometer-measured average sleep duration and sleep efficiency, self-reported insomnia symptoms, chronotype and daytime sleepiness, and clinically diagnosed sleep apnea based on ICD-10

Supplemental Table 2. Associations of accelerometer-measured sleep duration irregularity with incident cardiovascular events in the UK Biobank, excluding cases diagnosed in the first year of follow-up

	7-day sleep duration standard deviation					Per 1 hour	P-trend
	≤30 min	31-45 min	46-60 min	60-90 min	>90 min		
Major cardiovascular events							
Cases	303	613	520	401	203	2040	
Person-years	94060	191940	162456	121669	65916	636041	
Model 1	Ref	1.06 (0.92, 1.21)	1.14 (0.99, 1.31)	1.27 (1.09, 1.47)	1.24 (1.04, 1.48)	1.14 (1.06, 1.23)	0.001
Model 2	Ref	1.04 (0.91, 1.20)	1.10 (0.96, 1.27)	1.20 (1.03, 1.39)	1.17 (0.98, 1.40)	1.11 (1.03, 1.20)	0.01
Model 3	Ref	1.04 (0.91, 1.19)	1.10 (0.95, 1.26)	1.18 (1.02, 1.38)	1.13 (0.94, 1.36)	1.09 (1.01, 1.19)	0.03
Myocardial infarction							
Cases	164	299	271	199	111	1044	
Person-years	94431	192949	163240	122300	66232	639152	
Model 1	Ref	0.95 (0.79, 1.15)	1.09 (0.90, 1.32)	1.14 (0.92, 1.40)	1.21 (0.95, 1.54)	1.17 (1.05, 1.30)	0.004
Model 2	Ref	0.93 (0.77, 1.13)	1.04 (0.85, 1.26)	1.05 (0.85, 1.29)	1.11 (0.87, 1.41)	1.13 (1.01, 1.26)	0.03
Model 3	Ref	0.93 (0.77, 1.12)	1.02 (0.84, 1.24)	1.03 (0.84, 1.27)	1.05 (0.82, 1.34)	1.10 (0.98, 1.23)	0.1
Stroke							
Cases	147	322	260	211	101	1041	
Person-years	94495	192891	163410	122354	66359	639509	
Model 1	Ref	1.14 (0.94, 1.39)	1.18 (0.96, 1.44)	1.39 (1.13, 1.72)	1.31 (1.02, 1.69)	1.14 (1.02, 1.27)	0.02
Model 2	Ref	1.13 (0.93, 1.38)	1.16 (0.95, 1.42)	1.34 (1.09, 1.66)	1.26 (0.98, 1.63)	1.12 (1.00, 1.25)	0.04
Model 3	Ref	1.14 (0.93, 1.38)	1.16 (0.95, 1.42)	1.34 (1.08, 1.66)	1.25 (0.97, 1.63)	1.11 (0.99, 1.25)	0.06

Model 1: adjusted for age, sex, race, Townsend deprivation index, work schedules, and family history of CVD
Model 2: Model 1 + adjusted for BMI, smoking status, alcohol consumption, diet quality, physical activity, and history of hypertension, dyslipidemia, diabetes and depression
Model 3: Model 2 + adjusted for accelerometer-measured average sleep duration and sleep efficiency, self-reported insomnia symptoms, chronotype and daytime sleepiness, and clinically diagnosed sleep apnea based on ICD-10

Supplemental Table 3. Associations of accelerometer-measured sleep duration irregularity on weekdays with incident cardiovascular events in the UK Biobank

	7-day sleep duration standard deviation					Per 1 hour	P-trend
	≤30 min	31-45 min	46-60 min	60-90 min	>90 min		
Major cardiovascular events							
Cases	277	408	380	508	411	1984	
Person-years	84861	122075	111436	138939	96557	553867	
Model 1	Ref	1.00 (0.86, 1.17)	1.03 (0.88, 1.20)	1.09 (0.94, 1.26)	1.24 (1.07, 1.45)	1.18 (1.09, 1.28)	<.0001
Model 2	Ref	1.00 (0.85, 1.16)	1.01 (0.86, 1.17)	1.04 (0.90, 1.20)	1.15 (0.99, 1.35)	1.13 (1.04, 1.22)	0.003
Model 3	Ref	1.00 (0.85, 1.16)	1.00 (0.86, 1.17)	1.03 (0.89, 1.20)	1.14 (0.97, 1.33)	1.12 (1.03, 1.21)	0.005
Myocardial infarction							
Cases	141	203	178	263	205	990	
Person-years	85221	122592	111986	139666	97108	556573	
Model 1	Ref	0.99 (0.80, 1.23)	0.96 (0.77, 1.20)	1.13 (0.92, 1.39)	1.26 (1.02, 1.57)	1.19 (1.06, 1.32)	0.002
Model 2	Ref	0.98 (0.79, 1.21)	0.93 (0.74, 1.16)	1.06 (0.86, 1.30)	1.14 (0.92, 1.42)	1.11 (1.00, 1.24)	0.05
Model 3	Ref	0.98 (0.79, 1.22)	0.92 (0.74, 1.15)	1.05 (0.85, 1.29)	1.12 (0.90, 1.39)	1.10 (0.98, 1.23)	0.09
Stroke							
Cases	139	214	212	253	214	1032	
Person-years	85272	122666	111941	139716	97129	556725	
Model 1	Ref	1.04 (0.84, 1.29)	1.13 (0.91, 1.40)	1.06 (0.86, 1.30)	1.24 (1.00, 1.54)	1.18 (1.06, 1.31)	0.002
Model 2	Ref	1.03 (0.83, 1.28)	1.11 (0.90, 1.38)	1.02 (0.83, 1.26)	1.18 (0.95, 1.46)	1.14 (1.02, 1.27)	0.02
Model 3	Ref	1.03 (0.83, 1.28)	1.11 (0.90, 1.38)	1.02 (0.83, 1.26)	1.17 (0.94, 1.45)	1.14 (1.02, 1.27)	0.02

Model 1: adjusted for age, sex, race, Townsend deprivation index, work schedules, and family history of CVD
Model 2: Model 1 + adjusted for BMI, smoking status, alcohol consumption, diet quality, physical activity, and history of hypertension, dyslipidemia, diabetes and depression
Model 3: Model 2 + adjusted for accelerometer-measured average sleep duration and sleep efficiency, self-reported insomnia symptoms, chronotype and daytime sleepiness, and clinically diagnosed sleep apnea based on ICD-10

Supplemental Table 4. Associations of accelerometer-measured sleep duration irregularity with risk of ischemic and hemorrhagic stroke in the UK Biobank

	7-day sleep duration standard deviation					Per 1 hour	P-trend
	<30 min	30-44 min	45-59 min	60-89 min	≥90 min		
Ischemic stroke							
Cases	118	266	211	186	98	879	
Person-years	94519	192940	163443	122385	66379	639665	
Model 1	Ref	1.18 (0.95, 1.47)	1.20 (0.96, 1.50)	1.54 (1.22, 1.94)	1.59 (1.21, 2.08)	1.24 (1.10, 1.39)	0.0003
Model 2	Ref	1.17 (0.94, 1.46)	1.17 (0.93, 1.47)	1.47 (1.17, 1.86)	1.51 (1.16, 1.99)	1.21 (1.08, 1.36)	0.001
Model 3	Ref	1.18 (0.95, 1.46)	1.18 (0.94, 1.48)	1.48 (1.17, 1.87)	1.52 (1.15, 2.00)	1.21 (1.08, 1.37)	0.002
Hemorrhagic stroke							
Cases	44	105	81	55	24	309	
Person-years	94519	192940	163443	122385	66379	639665	
Model 1	Ref	1.23 (0.87, 1.75)	1.20 (0.83, 1.74)	1.18 (0.79, 1.76)	1.02 (0.62, 1.68)	0.97 (0.78, 1.21)	0.79
Model 2	Ref	1.22 (0.86, 1.74)	1.19 (0.82, 1.72)	1.15 (0.77, 1.72)	1.00 (0.61, 1.65)	0.96 (0.77, 1.20)	0.72
Model 3	Ref	1.21 (0.85, 1.73)	1.17 (0.81, 1.70)	1.13 (0.75, 1.68)	0.96 (0.58, 1.60)	0.94 (0.75, 1.18)	0.61

Model 1: adjusted for age, sex, race, Townsend deprivation index, work schedules, and family history of CVD
Model 2: Model 1 + adjusted for BMI, smoking status, alcohol consumption, diet quality, physical activity, and history of hypertension, dyslipidemia, diabetes and depression
Model 3: Model 2 + adjusted for accelerometer-measured average sleep duration and sleep efficiency, self-reported insomnia symptoms, chronotype and daytime sleepiness, and clinically diagnosed sleep apnea based on ICD-10

Supplementary Table 5. Subgroup analysis of the association between accelerometer-measured sleep duration irregularity and risk of myocardial infarction and stroke in the UK Biobank

	Myocardial infarction			Stroke		
	Cases/N	HR (95% CI)[1]	P-int	Cases/N	HR (95% CI)[1]	P-int
Age			0.04			0.27
<60 yrs	228/31903	1.02 (0.82, 1.27)		171/31903	1.02 (0.79, 1.32)	
≥60 yrs	955/54316	1.29 (1.16, 1.44)		1004/54316	1.20 (1.07, 1.34)	
Sex			0.87			0.04
Men	792/36651	1.21 (1.07, 1.36)		653/36651	1.27 (1.11, 1.44)	
Women	391/49568	1.26 (1.06, 1.49)		522/49568	1.04 (0.88, 1.23)	
Race			0.81			0.20
White	1144/83577	1.23 (1.12, 1.36)		1145/83577	1.18 (1.07, 1.31)	
Non-white	39/2642	1.14 (0.70, 1.86)		30/2642	0.76 (0.38, 1.54)	
Average sleep duration			0.46			0.0003
<7 hrs	483/29488	1.12 (0.99, 1.27)		416/29488	1.01 (0.87, 1.17)	
7-8 hrs	486/39890	1.24 (1.03, 1.50)		513/39890	1.26 (1.05, 1.51)	
>8 hrs	214/16841	1.34 (0.95, 1.87)		246/16841	1.73 (1.32, 2.27)	
Family history of CVD			0.005			0.75
No	363/34288	1.00 (0.82, 1.22)		405/34288	1.15 (0.97, 1.34)	
Yes	820/51931	1.33 (1.19, 1.49)		770/51931	1.18 (1.04, 1.34)	
PRS[2]			0.22			0.79
Low	198/28033	1.41 (1.14, 1.76)		318/28033	1.00 (0.80, 1.25)	
Medium	358/28034	1.29 (1.08, 1.54)		373/28035	1.34 (1.13, 1.58)	
High	599/28034	1.13 (0.98, 1.31)		456/28033	1.14 (0.96, 1.35)	

[1]Adjusted for age, sex, race, Townsend deprivation index, work schedules, and family history of CVD

[2]Among 84101 participants with genotyping information with additional adjustment of the first three genetic principal components

Supplemental Figure 1. Risk of incident MI (a) or stroke (b) according to joint categories of sleep duration irregularity and polygenic risk score for MI or stroke. Estimates adjusted for age, sex, race, Townsend deprivation index, work schedules, family history of CVD, and the first three genetic principal components.

(a) MI

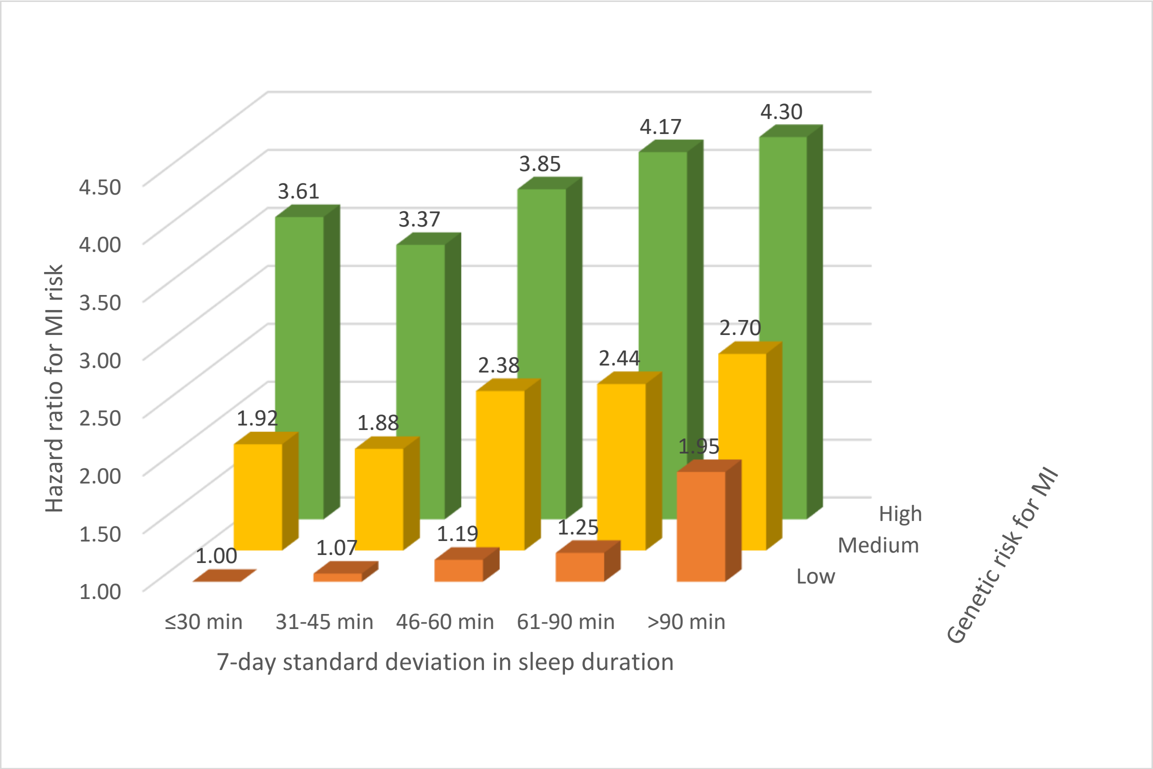

(b) Stroke

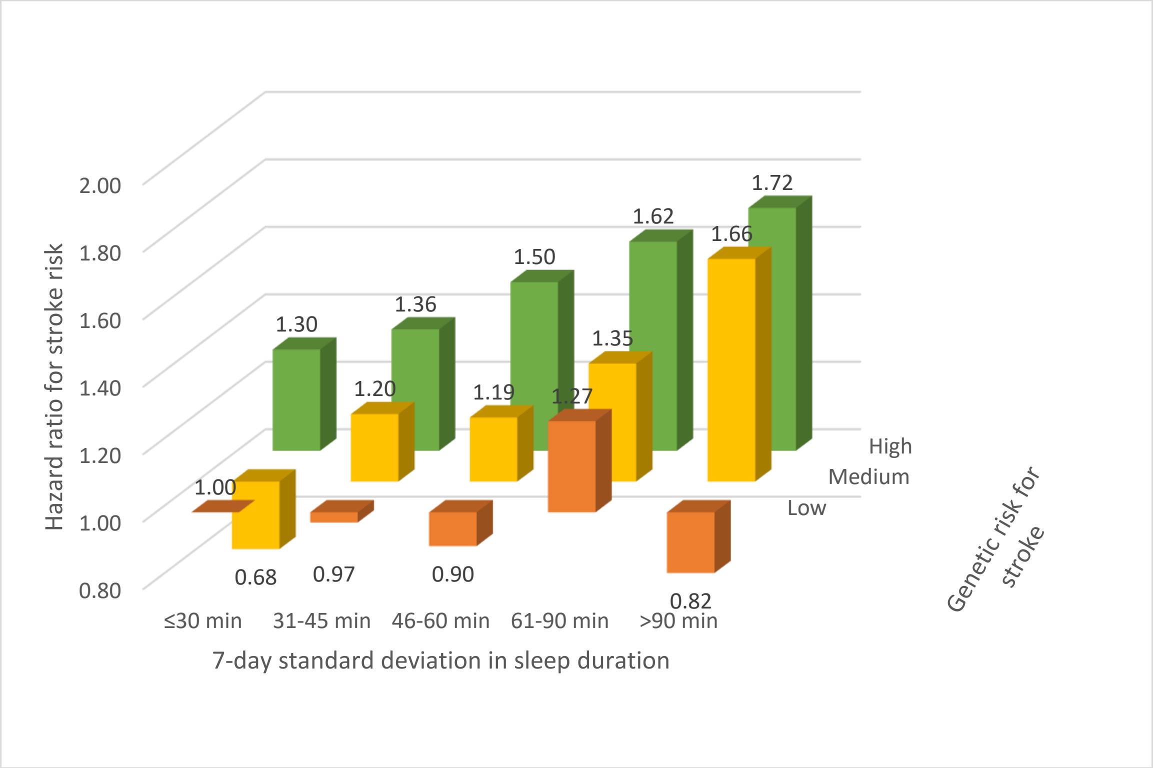

Hazard ratio for stroke risk
2.00
1.80
1.60
1.40
1.20
1.00
0.80
1.30
1.36
1.50
1.62
1.72
1.20
1.19
1.35
1.66
1.00
0.97
0.90
1.27
0.82
0.68
High
Medium
Low
Genetic risk for stroke
≤30 min
31-45 min
46-60 min
61-90 min
>90 min
7-day standard deviation in sleep duration

Supplemental Figure 2. Risk of incident MI (a) or stroke (b) according to joint categories of sleep duration irregularity and average sleep duration. Estimates adjusted for age, sex, race, Townsend deprivation index, work schedules, and family history of CVD.

(a) MI

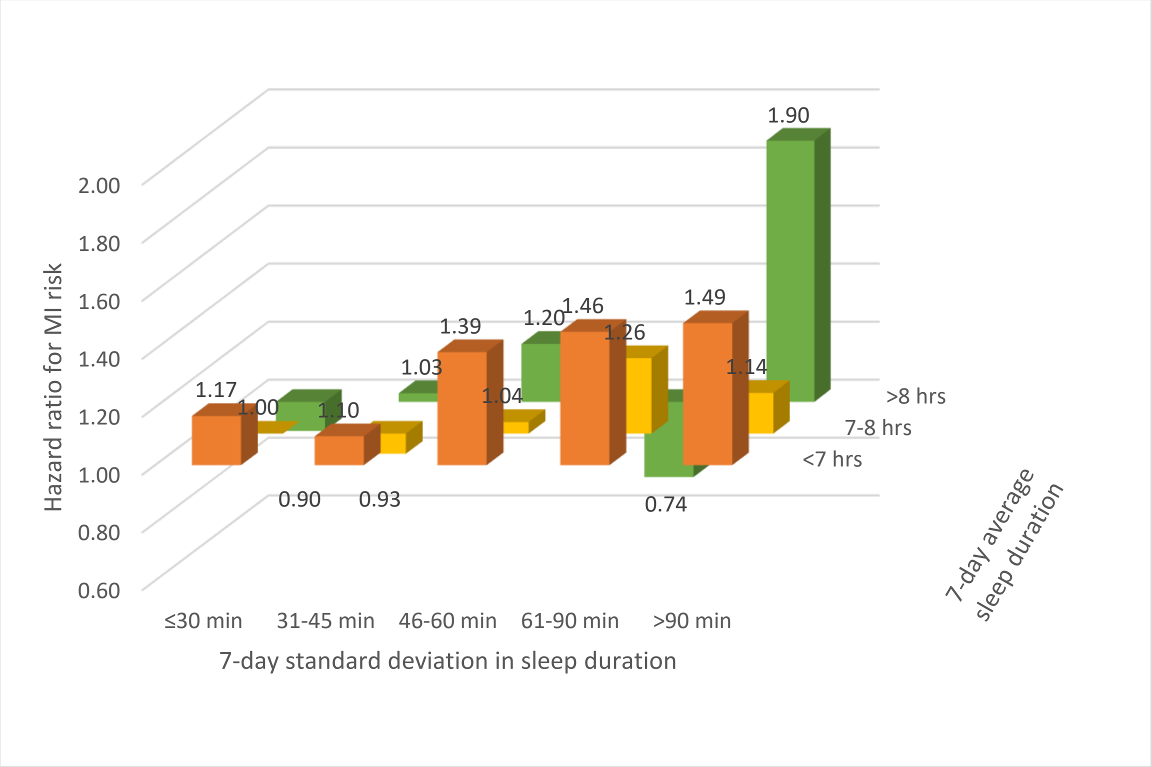

(b) Stroke

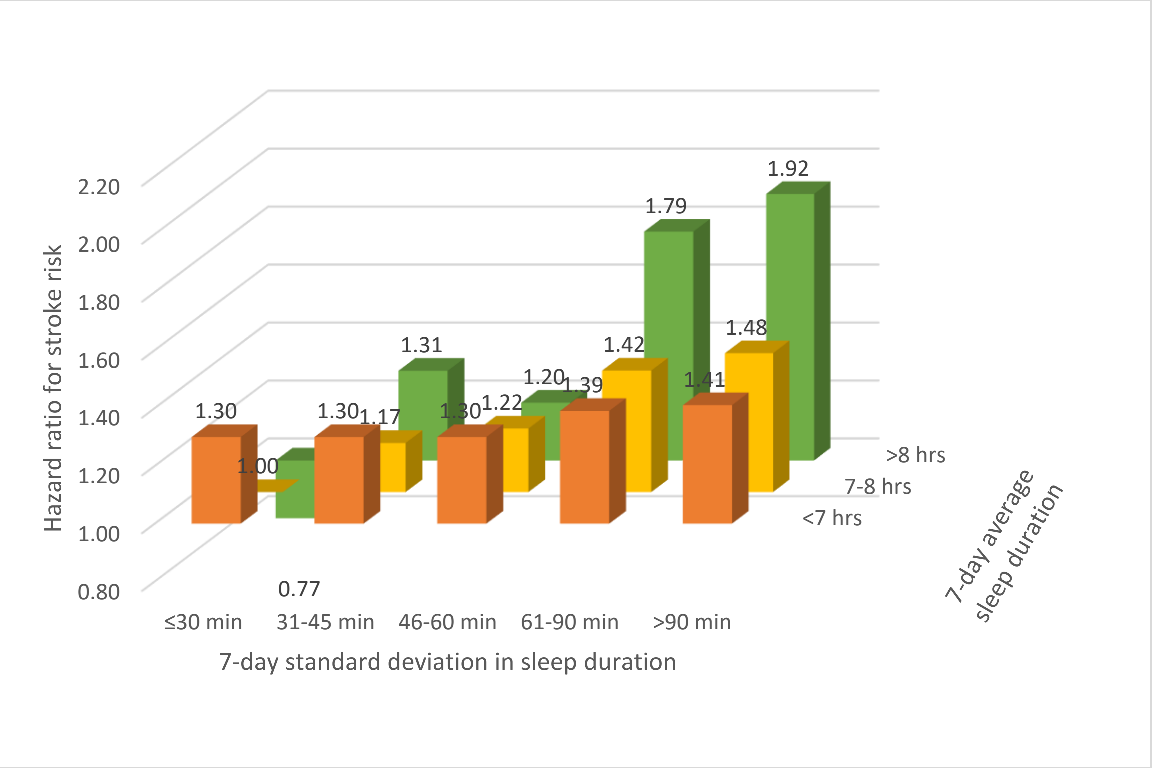

Hazard ratio for stroke risk
2.20
2.00
1.80
1.60
1.40
1.20
1.00
0.80
1.30
1.00
0.77
1.30
1.17
1.31
1.30
1.22
1.20
1.39
1.42
1.79
1.41
1.48
1.92
≤30 min
31-45 min
46-60 min
61-90 min
>90 min
7-day standard deviation in sleep duration
>8 hrs
7-8 hrs
<7 hrs
7-day average sleep duration
